# LTP in a Culture Dish

**DOI:** 10.1100/tsw.2001.47

**Published:** 2001-05-11

**Authors:** J.F. MacDonald, William Ju, Yu Tian Wang

**Affiliations:** Department of Physiology, University of Toronto, Canada

**Keywords:** AMPA receptors, NMDA receptors, synaptic plasticity, long-term potentiation, learning and memory

## Abstract

The “aging” of populations in the developed world is rapidly altering demographics and presents a number of challenges for science and medicine. Foremost among these challenges is the need to enhance the quality of life for this “aging” majority. Paradoxically, improved prevention and treatment of diseases will only increase the number of individuals who will lose quality of life because of cognitive deficits in learning and memory. Such cognitive deficits are particularly vexing in societies where the ability to deal with information technology has become an increasing necessity. Understanding how the human brain encodes and stores information becomes critical in designing required therapeutic strategies.

What are the molecular mechanisms of learning and memory? At the very least, an experimental model is required to address this question, and long-term potentiation (LTP) and/or long-term depression (LTD) of excitatory glutamatergic synaptic transmission in slices of the hippocampus form the most prevalent model of spatial leaning and memory [1-3]. A recent study [4] published in *Neuron* has unexpectedly shown that excitatory synapses between cultured hippocampal neurons can be more effectively used to monitor changes in postsynaptic receptor proteins that underlie LTP and LTD. At the vast majority of excitatory synapses the neurotransmitter L-glutamate is released and it activates postsynaptic AMPA (α-amino-3-hydroxy-5-methyl-4-isoxazoleproprionic acid) and NMDA (N-methyl-D-aspartic acid) ion channels (glutamate receptor proteins). AMPA channels are responsible for the largest component of the excitatory postsynaptic current (EPSC) while NMDA channels contribute less, due to their rapid and membrane potential-dependent block by extracellular $Mg^{2+}$. Nevertheless, NMDA receptors are permeable to $Ca^{2+}$ and this cation triggers a variety of biochemical events required for the altered synaptic strength [5]. At many synapses stimulus strength determines the form of the plasticity [1,2]. Paradoxically, NMDA receptor activation is often found to be required for either potentiation or depression.

LTP or LTD could result from changes in the amount of transmitter released, from changes in the number of active synapses, or from changes in the efficacy or number of postsynaptic receptors. Tremendous interest has focused upon recent demonstrations that LTD induces an increased rate of receptor internalization and a decrease in the number of synaptic AMPA receptors [6-9]. By corollary, could an increase in the number of AMPA receptors account for LTP? Certainly, there is strong evidence of increased delivery of GFP-tagged recombinant AMPA receptor subunits following the induction of LTP in hippocampal slices [10-12].

It would be highly desirable to have an experimental preparation, such as monolayer neuronal cultures, in which the insertion of synaptic AMPA receptors could be rigorously measured. However, demonstrating LTP at cultured synapses has often proved much more problematic than measuring LTD [13-14]. The study of Lu et al. [4] makes several methodological breakthroughs that permit the use of cultures to study LTP. Spontaneous mEPSCs, which result from the release of individual quanta of glutamate from presynaptic terminals, were measured in the absence of extracellular $Mg^{2+}$. However, the NMDA receptor component of mEPSCs was kept artificially small by leaving glycine, a co-agonist of this receptor, out of the bathing solution. In the absence of glycine, released glutamate cannot open NMDA channels. Subsequently glycine was transiently applied to the neuron for several minutes to cause a marked but transient enhancement of synaptically mediated NMDA receptor currents. Some NMDA receptors are also located at nonsynaptic sites but they would not be opened during applications of glycine because, by definition, they would not be exposed to synaptically released glutamate. This procedure was consistently followed by a long-lasting enhancement of the AMPA receptor component of mEPSCs that mimicked LTP in the slice preparation. For example, blocking NMDA receptors or preventing the induced rise of intracellular $Ca^{2+}$ prevented this potentiation. Paradoxically, a transient elevation of glycine paired with an exogenous application of NMDA always induced a long-term depression of the AMPA component. This was the case even when synaptic NMDA receptors were blocked beforehand with the use-dependent antagonist MK-801, suggesting that synaptic receptors caused LTP while extrasynaptic receptors caused depression. With this basic functional model the authors then used fluorescent immunocytochemical assays to quantify the number of AMPA receptors at the synapses before and following applications of glycine. The induction of LTP was correlated with increased surface expression of endogenous AMPA receptor GluR1 subunits in punctuate regions believed to be synapses. Both this increase in native GluR1 surface expression and the potentiation of mEPSCs were prevented by a tetanus toxin-induced cleavage of VAMP, a SNARE protein essential for vesicular exocytosis, and the delivery of new receptors to the synapses.

The novelty of the study of Lu et al. [4] arises from their development of a simple model of LTP in cultured neurons. Using this unique model the authors have shown that LTP is a property of activation of synaptic but not extrasynaptic NMDA receptors. This implies that the influx of $Ca^{2+}$ at postsynaptic spines activates different processes than at nonsynaptic sites. Although the processes following extrasynaptic NMDA receptor activation that lead to LTD may involve $Ca^{2+}$ influx induced, $InsP_3$ and ryanodine receptor-dependent $Ca^{2+}$ release from intracellular calcium store [15], and likely subsequent activation of calcineurin [16], the processes that link synaptic NMDA receptor activation to LTP remain largely unknown. Furthermore, this study provides the first evidence that membrane-fusion–dependent insertion of native AMPA receptors contributes to the enhanced synaptic transmission of LTP. This culture model of LTP shares a number of common features with stimulus-induced LTP in the hippocampus and the availability of this model should facilitate our efforts in understanding the molecular mechanisms underlying LTP.

| | | |
|---|---|---|
| Received | April | 27, 2001 |
| Accepted | April | 30, 2001 |
| Published | May | 11, 2001 |
